# Ammonia-based enrichment and long-term propagation of zone I hepatocyte-like cells

**DOI:** 10.1038/s41598-021-90708-3

**Published:** 2021-05-31

**Authors:** Ruri Tsuneishi, Noriaki Saku, Shoko Miyata, Saeko Akiyama, Palaksha Kanive Javaregowda, Kenta Ite, Nagisa Takashima, Masashi Toyoda, Tohru Kimura, Masahiko Kuroda, Atsuko Nakazawa, Mureo Kasahara, Hidenori Nonaka, Akihide Kamiya, Tohru Kiyono, Junji Yamauchi, Akihiro Umezawa

**Affiliations:** 1 Center for Regenerative Medicine, National Center for Child Health and Development Research Institute, 2-10-1 Okura, Setagaya, Tokyo, 157-8535 Japan; 2 Laboratory of Molecular Neuroscience and Neurology, Tokyo University of Pharmacy and Life Sciences, Hachioji, Tokyo, 192-0392 Japan; 3 Research Team for Geriatric Medicine (Vascular Medicine), Tokyo Metropolitan Institute of Gerontology, Tokyo, 173-0015 Japan; 4 Laboratory of Stem Cell Biology, Department of Biosciences, Kitasato University School of Science, Kanagawa, 252-0373 Japan; 5 Department of Molecular Pathology, Tokyo Medical University, 6-1-1 Shinjuku, Shinjuku-ku, Tokyo, 160-8402 Japan; 6 Saitama Children’s Medical Center, Saitama, 330-8777 Japan; 7 Organ Transplantation Center, National Center for Child Health and Development, Tokyo, 157-8535 Japan; 8 Department of Molecular Life Sciences, Tokai University School of Medicine, 143 Shimokasuya, Isehara, Kanagawa 259-1193 Japan; 9 Project for Prevention of HPV-Related Cancer, Exploratory Oncology Research and Clinical Trial Center, National Cancer Center, Chiba, 277-8577 Japan

**Keywords:** Cell biology, Stem cells

## Abstract

Ammonia has a cytotoxic effect and can therefore be used as a selection agent for enrichment of zone I hepatocytes. However, it has not yet been determined whether ammonia-treated hepatocyte-like cells are able to proliferate in vitro. In this study, we employed an ammonia selection strategy to purify hepatocyte-like cells that were differentiated from human embryonic stem cells (ESCs) and from induced pluripotent stem cells (iPSCs). The resistance to cytotoxicity or cell death by ammonia is likely attributable to the metabolism of ammonia in the cells. In addition to ammonia metabolism-related genes, ammonia-selected hepatocytes showed increased expression of the cytochrome P450 genes. Additionally, the ammonia-selected cells achieved immortality or at least an equivalent life span to human pluripotent stem cells without affecting expression of the liver-associated genes. Ammonia treatment in combination with in vitro propagation is useful for obtaining large quantities of hepatocytes.

## Introduction

Hepatocytes have been used as a substitute for animals in preclinical safety tests that investigate the hepatotoxicity of low-molecular weight drugs^1^. Primary culture of hepatocytes dissociated from a liver is the gold standard in pharmaceutical in vitro studies for clinical prediction. The drawbacks of the use of hepatocytes in drug screening include limited supply of a given lot and large variations between lots due to genetic and environmental backgrounds. To solve the issue of lot variation, HepG2 cells are also used to examine hepatotoxicity because of their clonal nature. HepaRG, another hepatocyte-like clone, was established from a hepatoblastoma and has the advantage of highly inducible cytochrome P450 genes^2^. In addition to isolated hepatocytes, immortalized hepatocytes, and hepatocarcinoma cells, hepatocytes or hepatocyte-like cells can be differentiated from human pluripotent stem cells (PSCs) such as embryonic stem cells (ESCs) and induced pluripotent stem cells (iPSCs). Human PSCs have impacted numerous medical research fields including clinical therapy development, drug discovery, research on inherited diseases and studies on reprogramming of differentiated cells^3–6^. For example, human PSC-derived hepatocytes serve as an in vitro tool for understanding drug metabolism and toxicology^7,8^. Human PSC-derived hepatocytes or hepatocyte-like cells can be obtained from the same origin repeatedly due to the immortality of ESCs^9–11^. Parenchymal hepatocytes in liver lobules have different characteristics and functions in lobular zones: Zone I hepatocytes are found around portal area, zone III hepatocytes are around central vein, and zone II hepatocytes are in an intermediate location. Zone I hepatocytes have metabolic activity towards ammonia and perform glycogenesis, while zone III hepatocytes show drug metabolic activity and perform glycolysis. These zones are believed to depend on concentrations of oxygen and hormones. Hepatocytes can be selected by ammonia because hepatocytes actively metabolize ammonia^12^. Likewise, hepatocyte-like cells differentiated from human PSCs can be enriched with ammonia^13^. Hepatocytes can be maintained in vitro without the introduction of any genes^14,15^. In this study, we used drug-induced liver injury (DILI)-derived iPSCs and ESCs as diseased cells and healthy pluripotent cells, respectively, to determine the potential application of the DILI-derived cells for drug toxicity testing. We propagated hepatocytes or hepatocyte-like cells derived from human PSC, i.e. ESCs and iPSCs, and enriched for zone I hepatocytes with ammonia^16,17^. Ammonia-selected cells proliferated in vitro at least to 30 population doublings (PDs) over a span of more than 190 days. This study introduces an alternative strategy to obtain large numbers of human hepatocytes by a combination of ammonia selection and subsequent in vitro propagation.

## Results

### Selection of ESC-derived hepatocyte-like cells with ammonia

**Figure 1:**
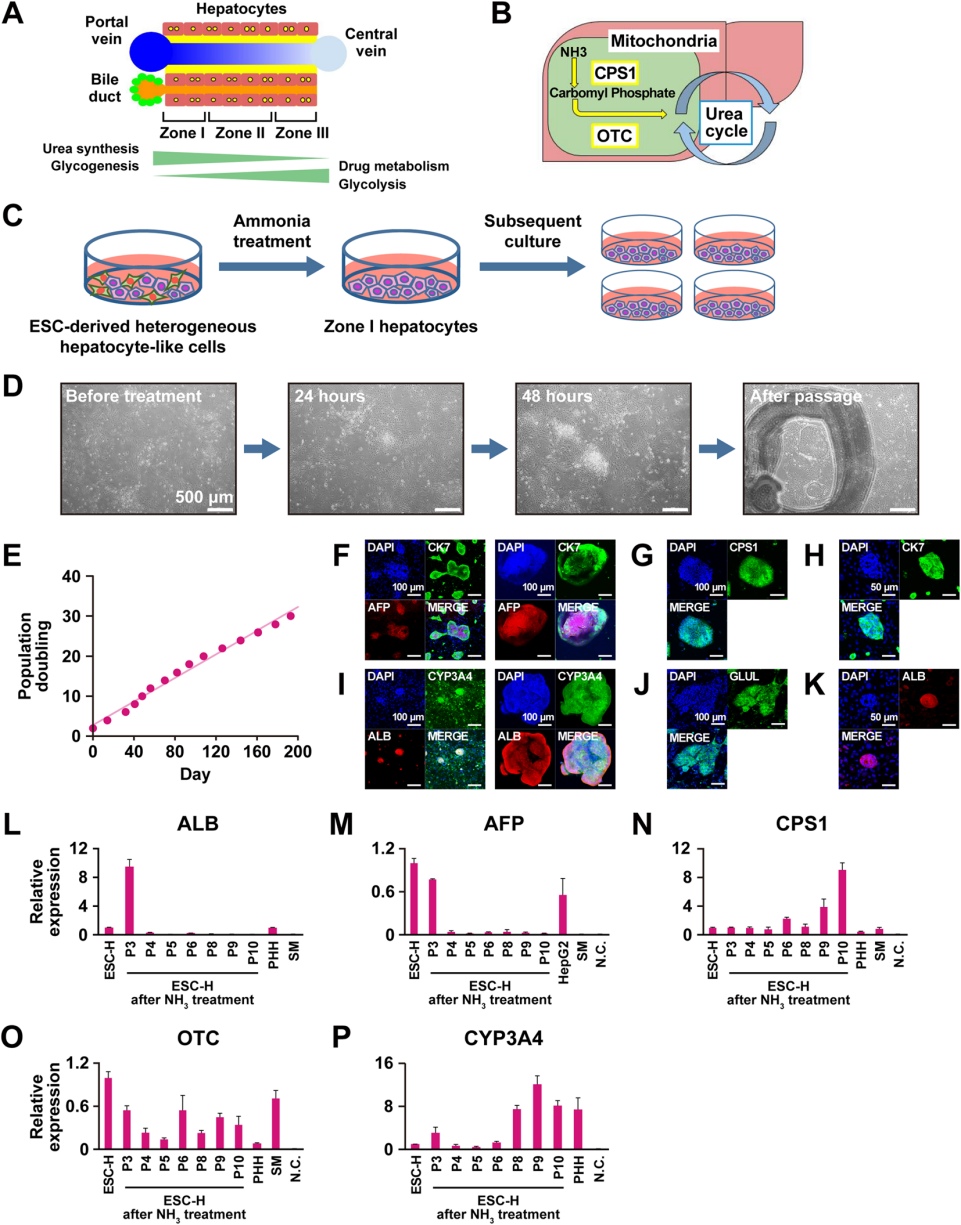
Generation of hepatocyte-like cells from SEES2 ESCs after exposure to ammonia. (**A**,**B**) Hepatocytes in liver that metabolize ammonia. (**C**) Scheme for characterization of ammonia-selected hepatocyte-like cells and ammonia metabolism. (**D**) Phase-contrast photomicroscopy of the hepatocyte-like cells at passage 3 during the selection with 0.02% ammonia. Panels shown in (**D**) are: Cells without treatment (before treatment), cells treated for 24 h (24 h), cells treated for 48 h (48 h) and passaged cells (after passage). Bar: 500 μm. (**E**) Growth curve of the ammonia-selected cells. (**F**) Immunocytochemistry of the ammonia-selected cells by using antibodies to CK7 and AFP. (**G**) Immunocytochemistry of the ammonia-selected cells using an antibody to CPS1. (**H**) Immunocytochemistry of the ammonia-selected cells using an antibody to CK7. (**I**) Immunocytochemistry of the ammonia-selected cells by using antibodies to CYP3A4 and ALB. (**J**) Immunocytochemistry of the ammonia-selected cells using an antibody to GLUL. (K) Immunocytochemistry of the ammonia-selected cells using an antibody to ALB. (**L**–**P**) qRT-PCR analysis of the genes for ALB (**L**), AFP (**M**), CPS1 (**N**), OTC (**O**) and CYP3A4 (**P**). Each expression level was calculated from the results of technical triplicate experiments. Average fold increase with standard deviation is shown. From left to right: ESC-derived hepatocyte-like cells without ammonia selection, ammonia-selected ESC-derived hepatocyte-like cells at passage 3–10. HepG2 cells, negative control (H_2_O), iPSC-derived immortalized cells (SM).

To obtain zone I hepatocytes, SEES2 ESCs were differentiated into hepatocyte-like cells (Fig. 1A–D). After differentiation, this heterogenous population of ESC-derived hepatocyte-like cells was exposed to ammonia for 2 days, which kills most cells. The surviving ammonia-selected cells proliferated as colonies and exhibited hepatocyte-like morphology.

### Long-term cultivation of ESC-derived hepatocyte-like cells

The cells that survived after exposure to ammonia retained replicative capacity on an mouse embryonic fibroblast (MEF) feeder layer. The ammonia-selected cells continued to proliferate in vitro at for least up to 30 PDs over a span of more than 190 days (Fig. 1E). Immunocytochemistry revealed that the ammonia-selected cells were positive for AFP, ALB, CYP3A4, CPS1, GLUL, and CK7 (Fig. 1F–K). Immunoreactivity of the ammonia-selected cells at the central and peripheral portions of the colonies stained positive for AFP and CK7, respectively (Fig. 1F). The cells at the peripheral portion of colonies stained positive for ALB, and almost all cells in the colonies stained positive for CYP3A4 (Fig. 1I). In certain colonies, all cells in the colonies were positive for CK7 and ALB (Fig. 1H,K). These results imply that the ammonia-selected cells retained hepatic and ductal characteristics after more than 30 PDs. The ammonia-selected hepatocyte-like cells were then subjected to qRT-PCR analysis to investigate expression levels of hepatocyte-associated genes (Fig. 1L–P). After 3 passages, the ammonia-selected cells expressed the genes for ALB, AFP, CYP3A4, CPS1, and OTC. The expression levels of the genes for ALB and AFP decreased, but those of the genes for CYP3A4 and CPS1 increased dramatically during serial passaging. The increased CPS1 expression may be attributed to an increased population of cells with CPS1 expression. Expression levels of the OTC gene remained unchanged upon passaging. In addition, urea production by the hepatocyte-like cells was measured. The urea production of the hepatocyte-like cells at passage 7 and 17 was 20.6 (± 0.01) and 9.4 (± 0.00) μg/day/million cells, respectively.

### Feeder cells for in vitro proliferation of ESC-derived hepatocyte-like cells

**Figure 2:**
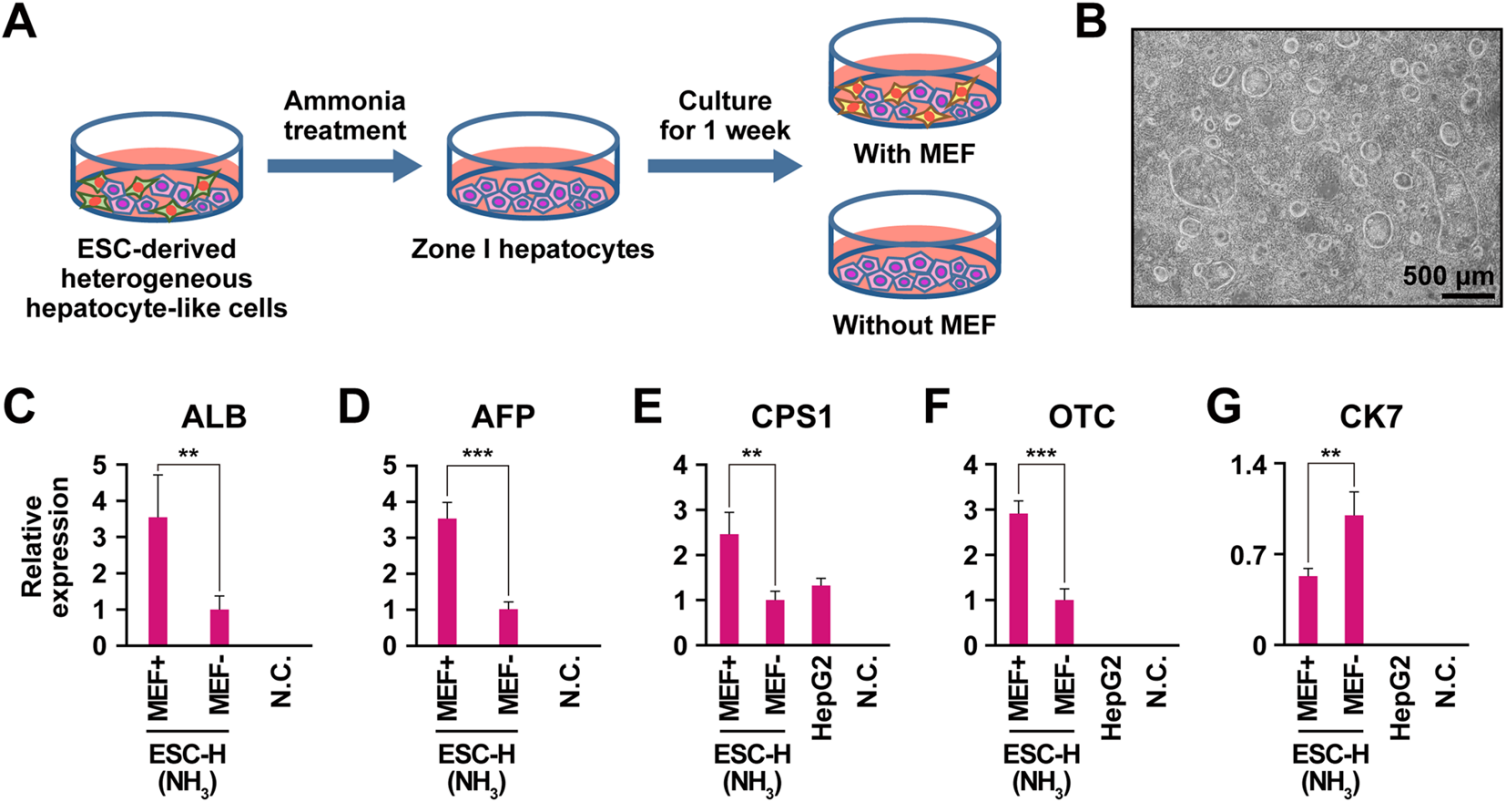
Requirement of feeder cells to support in vitro proliferation of the ammonia-selected hepatocyte-like cells. (**A**) Scheme for feeder cell dependency of ammonia-selected hepatocyte-like cells. (**B**) Phase contrast photomicrograph of the ammonia-selected hepatocyte-like cells on mouse embryonic fibroblasts (MEFs). (**C**–**G**) qRT-PCR analysis of the genes for ALB (**C**), AFP (**D**), CPS1 (**E**), OTC (**F**) and CK7 (**G**). Each expression level was calculated from the results of technical triplicate experiments. Average fold increase with standard deviation is shown. Statistical analysis was performed using the unpaired two-tailed Student's t test. **P < 0.01, ***P < 0.001. Irradiated MEFs and diethylpyrocarbonate (DEPC)‑treated water were used for negative controls in each experiment. Irradiated MEFs and DEPC-treated water did not express the genes for ALB, AFP, CPS1, OTC and CK7.

Proliferation of the ESC-derived ammonia-selected cells was dependent on MEF feeder cells. The ammonia-selected cells detached from dishes without the MEF feeders within 2 weeks. We therefore compared MEF feeder and feeder-free conditions (Fig. 2A). The ammonia-selected cells steadily proliferated on the MEF feeder layer (Fig. 2B). Expression levels of the genes for ALB, AFP, CPS1, and OTC were significantly higher on the MEF feeder than under feeder-free conditions (Fig. 2C–F). On the contrary, expression of the CK7 gene decreased on the MEF feeder (Fig. 2G).

### Spheroid formation of the ESC-derived hepatocyte-like cells for maturation

**Figure 3:**
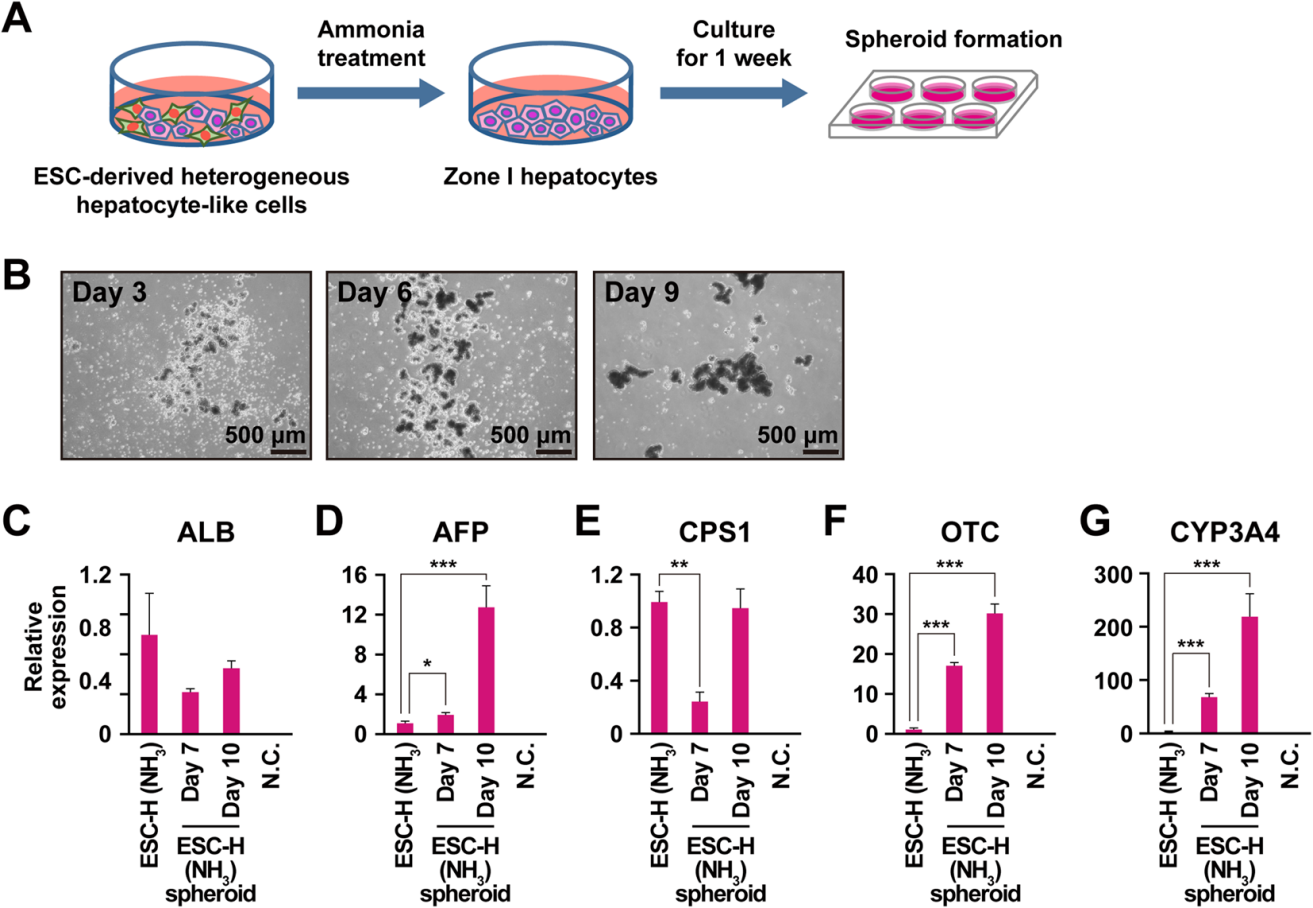
Up-regulation of the genes for ammonia metabolism via three-dimensional cultivation in the ammonia-selected hepatocyte-like cells. (**A**) Protocol for spheroid formation of ammonia-selected hepatocyte-like cells. (**B**) Phase-contrast photomicrograph of the ammonia-selected hepatocyte-like cells at 3, 6, and 9 days after spheroid formation. (**C**–**G**) qRT-PCR analysis of the genes for ALB (**C**), AFP (**D**), CPS1 (**E**), OTC (**F**) and CYP3A4 (**G**). Each expression level was calculated from the results of technical triplicate experiments. Average fold increase with standard deviation is shown. Statistical analysis was performed using one-way ANOVA, followed by Dunnett's multiple comparisons test. *P < 0.05, **P < 0.01, ***P < 0.001. From left to right: Ammonia-selected hepatocyte-like cells at passage 12 without sphere formation, ammonia-selected hepatocyte-like cells at passage 12 with sphere formation (Day 7, Day 10), HepG2 cells, negative control (H_2_O).

We investigated hepatocytic maturation through spheroid formation using the ammonia-selected cells. We employed a three-dimensional (3D) culture method to form spheroids (Fig. 3A). Spheroids formed on day 3, increased in size on day 6, and appeared unchanged on Day 9 (Fig. 3B). The genes for AFP, OTC and CYP3A4 were significantly up-regulated in the cells after spheroid formation (Fig. 3C–G).

### Selection of iPSC-derived hepatocyte-like cells with ammonia

**Figure 4:**
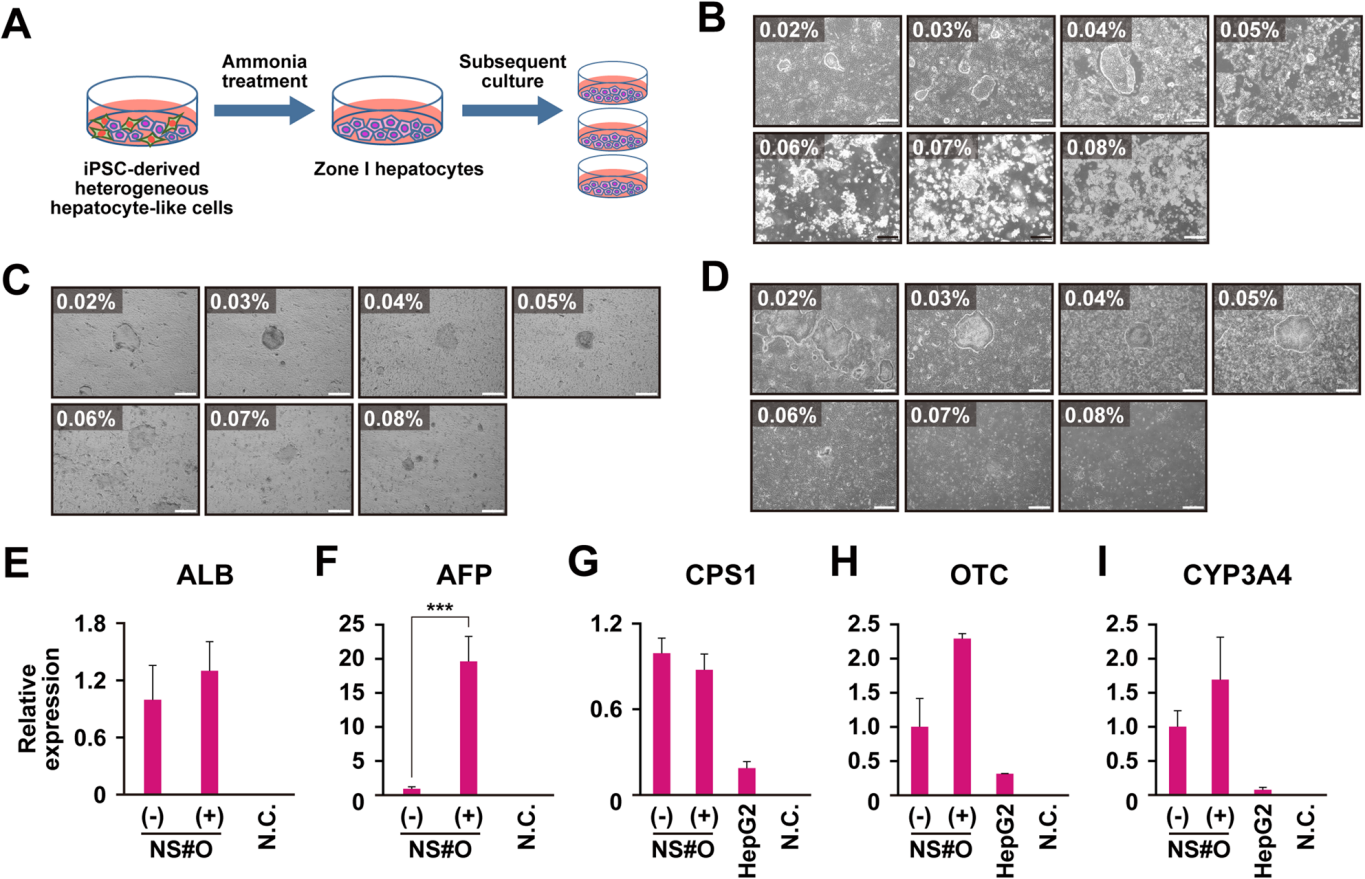
Ammonia selection of iPSC-derived hepatocyte-like cells. (**A**) Scheme for ammonia selection of iPSC-derived hepatocyte-like cells. (**B**–**D**) Phase contrast photomicrograph of the hepatocyte-like cells generated from DILI-derived iPSC-O cells exposure to the indicated concentration of ammonia at day 0 (**B**), and with exposure to the indicated concentration of ammonia for 1 day (**C**) or 2 days (**D**). (**E**–**I**) qRT-PCR analysis of the genes for ALB (**E**), AFP (**F**), CSP1 (**G**), OTC (**H**) and CYP3A4 (**I**). Each expression level was calculated from the results of technical triplicate experiments. Average fold increase with standard deviation is shown. Statistical analysis was performed using the unpaired two-tailed Student's t test. ***P < 0.001. From left to right: Hepatocyte-like cells generated from DILI-derived iPSC-O cells without ammonia selection, ammonia-selected hepatocyte-like cells generated from DILI-derived iPSC-O cells, HepG2 cells, negative control (H_2_O).

iPSCs were examined to determine their potency to differentiate into hepatocytes. We used iPSC-O iPSCs from a patient with drug-induced hepatic injury (DILI-iPSCs) for hepatic differentiation. iPSC-O cells were exposed to ammonia for 2 days after they were differentiated into heterogenous population of differentiated cells (Fig. 4A). The differentiated cells, including hepatocyte-like cells, were treated with ammonia in three independent experiments (Fig. 4B–D). The cell-killing effect of ammonia depended on the ammonia concentration. After exposure to ammonia, the selected cells exhibited hepatocyte-like morphologies such as formation of epithelial monolayers, flatter cells, a large cytoplasmic-to-nuclear ratio, numerous and prominent nucleoli, and occasional binucleated cells. These hepatocyte-like cells were propagated in vitro and examined for expression of the hepatocyte-associated genes (Fig. 4E–I). The iPSC-O differentiated cells after ammonia selection showed 2.1-, 20.1-, and 3.0-fold increased expression of the genes for ALB, AFP, and OTC, respectively. Likewise, the iPSC-O differentiated cells after ammonia selection increased the gene for CYP3A4. The gene chip analysis showed that SEES2 cell-derived and iPSC-O cell-derived hepatocyte-like cells exhibited increased expression of the genes for cytochrome P450 and phase 2 enzymes upon exposure to ammonia (Supplementary Fig. 1).

## Discussion

### Ammonia-based enrichment of zone I hepatocytes

Ammonia has a cell-killing effect, though the mechanism remains unclear. Ammonium ions compete with potassium ions for inward transport over the cytoplasmic membrane, via potassium transport proteins like the Na^+^/K^+^ ATPase and the Na^+^K^+^2Cl-cotransporter. This competition of ammonia with potassium ions lead to predictable intracellular and/or extracellular pH changes and subsequent cell death. Neural toxicity of ammonia is associated with intracellular pH change through inhibition of potassium channel and depolarization of GABA neurons^18,19^. Hepatocytes predictably have high metabolic activity towards ammonia and can evade its cytotoxic effects^12^. The escape of PSC-derived differentiated cells from ammonia is therefore explained by endogenous ammonia metabolism, i.e. expression of urea cycle-related genes such as OTC and CPS1^13^. To enrich for cells that can metabolize ammonia, selection with cell surface markers such as CD13 by flow cytometric analysis and magnetic cell sorting can be used. Introduction of cell type-specific expression of cytotoxic antibiotic-resistant genes such as the neomycin-resistant gene is also available for cell selection^20^. Compared with these sophisticated approaches, successful enrichment with ammonia exposure as used in this study is simple and straightforward. Indeed, the ammonia selection method was also applicable to the HepaMN immortalized cell line^21^. It is also noteworthy that ammonia selection may increase homogeneity or decrease heterogeneity in a cell lot with regard to ammonia metabolic activity.

### Altered and unaltered gene expression after ammonia selection and during propagation

The expression levels of hepatocyte-associated genes such as the CPS1 and CYP3A4 genes gradually increased during the long-term cultivation of ammonia-selected hepatocyte-like cells. CPS1, an ammonia-metabolizing gene, was likely up-regulated after ammonia exposure and subsequent propagation because the increased expression might correlate with ammonia metabolic activity. In contrast, upregulation of the cytochrome P450 gene after ammonia selection and subsequent propagation was unexpected. This gene is expressed in zone III hepatocytes^22^ which are involved in drug metabolism. This increase of cytochrome P450 gene expression may be explained by an increased number of PSC-derived zone I hepatocytes with expression of the cytochrome P450 gene. Another possibility is that the ammonia-selected zone I hepatocytes express cytochrome P450 genes during long-term cultivation of PSC-derived cells. Along with the increase of the cytochrome P450 genes, ALBUMIN gene expression decreased during cultivation. The reciprocal changes in gene expression may also be attributable to population changes in cell type or changes in gene expression in the cells. These changes in gene expression are not correlated with physiological conditions or developmental processes but could be an artifact of some in vitro event. However, the cells that efficiently metabolize ammonia and show high CYP3A4 expression after more than 200 days in culture are available for use in pharmacokinetic/pharmacodynamic and toxicology testing, and can also be used as a model of regenerative therapy products.

### Effect of three-dimensional organoid formation on maturation

Maturation procedures for hepatocytes and hepatocytic progenitors include exposure of the cells to low molecular weight molecules such as dexamethazone and demethylsulfoxide, and cytokines such as Oncostatin M and hepatocyte growth factor. Cultivation in B27 medium is also used for maturation. Three-dimensional organoid formation enhanced up-regulation of the genes for ALB, AFP and OTC but Oncostatin M and hepatocyte growth factor had a little effect on maturation. Spheroids are difficult to use for toxicology testing because they float in the culture medium, but they are preferred for regenerative therapy products because of their high levels of hepatocyte-associated markers.

### Immortality or limited replicative capacity

Human primary cells in culture exposed to “culture stress” inevitably accumulate the p16INK4A protein, which acts as a negative regulator of the cell cycle by inhibiting the cyclin D-CDK4 kinase complex, resulting in activation of retinoblastoma protein (pRB) and cell cycle arrest^23,24^. Exogenous expression of an R24C mutant of CDK4, which cannot be bound by the p16INK4A protein and cyclin D1 therefore induces constitutive activation of the cyclin D-CDK4 complex, and induces pRB phosphorylation, resulting in escape from premature senescence. In this study, we are not able to determine whether the ammonia-selected hepatocyte-like cells derived from both ESCs and iPSCs have a finite or infinite life spans. However, the life span of the ammonia-selected cells, up to 30 population doublings over the course of 200 days, which is enough to obtain sufficient quantities of raw materials for drug development and pharmacokinetic, toxicology and regenerative therapy studies. Proof of immortality may require more than 100 population doublings, and this may possibly be achieved by continuous cultivation for about 2 years, based on a cell growth curve (Fig. 1E). Human hepatocytes are difficult to propagate ex vivo due to lack of appropriate cultivation conditions. To solve this problem, hepatocytes or hepatocyte-like cells have been obtained from normal livers, hepatomas/hepatocarcinomas, and ESCs/iPSCs^25–28^. Large-scale preparation of hepatocytes or hepatocyte-like cells with specific activities can be achieved with the three approaches: (1) in vitro propagation of cells with hepatocytic features, (2) maturation of propagated endodermal progenitors from PSCs, (3) hepatic differentiation of propagated PSCs.

### Possible regenerative therapy using ammonia-selected cells

Liver progenitor cells exist in the adult liver and can proliferate in order to preserve hepatic function in vitro and contribute to tissue maintenance and repair^12^. The usage of intrinsic hepatocyte progenitors provides a simple and efficient way to supply functional hepatocytes for clinical applications. This strategy has been used to produce cells for a human clinical trial^25^. Likewise, ammonia selection and enrichment in combination with generation and differentiation/maturation of human PSCs is a robust and cost-effective strategy. Human cells generated from PSCs with high levels of ammonia-metabolizing enzymes may be a new source of cell therapy products. Patients with congenital metabolic disorders such as urea cycle disorder and citrullinemia suffer from hyperammonemia^25^, which are caused by mutation or deletion of genes related to ammonia metabolism. Ammonia-selected cells with a high metabolic activity towards ammonia may be a potential treatment for these patients through an appropriate implantation strategy.

## Experimental procedures

### Ethical statement

All experiments handling human cells and tissues were approved by the Institutional Review Board at the National Center for Child Health and Development. Informed consent was obtained from all participants. When participants were under 18, informed consent was obtained from parents. Human cells in this study were utilized in full compliance with the Ethical Guidelines for Medical and Health Research Involving Human Subjects (Ministry of Health, Labor, and Welfare (MHLW), Japan; Ministry of Education, Culture, Sports, Science and Technology (MEXT), Japan). The derivation and cultivation of ESC lines were performed in full compliance with “the Guidelines for Derivation and Distribution of Human Embryonic Stem Cells (Notification of MEXT, No. 156 of August 21, 2009; Notification of MEXT, No. 86 of May 20, 2010) and “the Guidelines for Utilization of Human Embryonic Stem Cells (Notification of MEXT, No. 157 of August 21, 2009; Notification of MEXT, No. 87 of May 20, 2010)”. Animal experiments were performed in compliance with the basic guidelines for the conduct of animal experiments in implementing agencies under the jurisdiction of the Ministry of Health, Labour and Welfare (Notification of MHLW, No. 0220-1 of February 20, 2015). The protocols of the animal experiments were approved by the Institutional Animal Care and Use Committee of the National Research Institute for Child Health and Development. This study was carried out in compliance with the ARRIVE guidelines.

### Culture of ESCs

SEES2 ESCs were routinely cultured on a feeder layer of freshly plated gamma-irradiated mouse embryonic fibroblasts (MEFs) isolated from ICR embryos at 12.5 d gestation and passaged 2 times before irradiation (30 Gy), in the ESC culture media^16,17^. The ESC media consisted of KNOCKOUT-Dulbecco’s modified Eagle’s medium (KO-DMEM) (Life Technologies, CA, USA; #10829-018) supplemented with 20% KNOCKOUT-Serum Replacement (KO-SR; #10828-028), 2 mM Glutamax-I (#35050-079), 0.1 mM non-essential amino acids (NEAA; #11140-076), 50 U/ml penicillin-50 μg/ml streptomycin (Pen-Strep) (#15070-063), 0.055 mM β-mercaptoethanol (#21985-023) and recombinant human full-length bFGF (#PHG0261) at 10 ng/ml (all reagents from Life Technologies).

### Culture of iPSCs

Human cells were isolated from surplus liver tissue of a one-year-old girl with fulminant hepatitis (Hep2064). Primary cells were isolated according to the collagenase perfusion method^25,29,30^. iPSC-O cells were generated by introduction of Sendai virus carrying the 4 Yamanaka factors^31^. The iPSC-O cells were cultured on MEFs and grown using a medium for human ES cells [KNOCKOUT DMEM (Gibco, 10829), 15% CTS KNOCKOUT SR XenoFree Medium (Gibco, 14150), 1% penicillin streptomycin (Gibco, 11140), 1% non-essential amino acids (Gibco, 11360), 1% Sodium Pyruvate (Gibco, 11360), 1% Gulta MAX (Gibco, 35050), 0.1% β-mercaptoethanol (Gibco, 21985-023), and 10.0 ng/ml bFGF (INVITROGEN, PHG0024)].

### Preparation of feeder cells

MEFs were prepared for use as nutritional support (feeder) cells. Heads, limbs, tails, and internal organs were removed from E12.5 ICR mouse fetuses (Japan CLEA), minced with a blade, and seeded into culture dishes in a medium containing 10% FBS, 1% Pen-Strep to allow cell growth, and 1/100 (v/v) of 1 M HEPES buffer solution (INVITROGEN, 15630-106) was added to the cells. Following irradiation with an X-ray irradiation apparatus (Hitachi, MBR-1520 R-3; dose: 30 Gy), the cells were frozen using a TC protector (DS Pharma Biomedical, TCP-001).

### Preparation of primary human hepatocytes

Healthy human hepatocytes were isolated from surplus liver tissue of a 35-year-old healthy female according to the collagenase perfusion method^25,29,30^. First, liver tissues were shredded and washed with HEPES buffer (pH 7.7; 140 mM NaCl/2.68 mM KCl/0.2 mM Na_2_HPO_4_/10 mM HEPES). The tissues were then treated with 0.5 mg/mL collagenase/DMEM (Boehringer Mannheim) and diluted in the same buffer supplemented with 0.075% CaCl_2_ at 37 °C with gentle agitation. Cells were washed twice with HEPES buffer and then with 10% FBS, hEGF, transferrin, hydrocortisone, BSA, ascorbic acid, fungizone, 100 μg/mL Pen-Strep, 5 μg/mL insulin, and 5 × 10^–7^ M hydrocortisone hemisuccinate. The isolated cells were washed with 100 μg/mL Pen-Strep. The isolated cells were resuspended in HCM BULLETKITmedium (Cat:CC-3198, LONZA) supplemented with hydrocortisone hemisuccinate and frozen for future use. The cells were thawed and cultured in ESTEM-HE medium (GlycoTechnica, Ltd., Japan) to obtain primary human hepatocytes.

### Hepatocytic differentiation

To generate embryoid bodies (EBs), ESCs and iPSCs (1 × 10^4^/well) were dissociated into single cells with Accutase (Thermo Scientific, MA, USA) after exposure to the rock inhibitor (Y-27632: A11105-01, Wako, Japan), and cultivated in the 96-well plates in the EB medium [76% KNOCKOUT DMEM, 20% KNOCKOUT Serum Replacement (Life Technologies, CA, USA), 2 mM GlutaMAX-I, 0.1 mM NEAA, Pen-Strep, and 50 µg/mL l-ascorbic acid 2-phosphate (Sigma-Aldrich, St. Louis, MO, USA)] for 10 days. The EBs were transferred to the 24-well plates coated with collagen type I, and cultivated in the XF32 medium [85% KNOCKOUT DMEM, 15% KNOCKOUT Serum Replacement XF CTS (XF-KSR; Life Technologies), 2 mM GlutaMAX-I, 0.1 mM NEAA, Pen-Strep, 50 µg/mL l-ascorbic acid 2-phosphate (Sigma-Aldrich, St. Louis, MO, USA), 10 ng/mL heregulin-1β (recombinant human NRG-β 1/HRG-β1 EGF domain; R&D Systems, Minneapolis, MN, USA), 200 ng/mL recombinant human IGF-1 (LONG R^3^-IGF-1; Sigma-Aldrich), and 20 ng/mL human bFGF (Life Technologies)] for 14 to 35 days. The differentiated cells were further cultivated and propagated in ESTEM-HE medium (GlycoTechnica, Ltd., Japan) containing Wnt3a and R-spondin 1 at 37 °C in a humidified atmosphere containing 95% air and 5% CO_2_^32,33^. When the cultures reached subconfluence, the cells were harvested with Trypsin–EDTA solution (cat#23315, IBL CO., Ltd, Gunma, Japan), and re-plated at a density of appropriately 5 × 10^5^ cells in a 100-mm dish. Medium changes were carried out three times a week thereafter.

We also used HepaMN and SM cells for qRT-PCR analysis. HepaMN cells have been established from a liver associated with biliary atresia^21^. SM cell has been generated as an immortalized cell from a patient with drug-induced liver injury (Supplementary Experimental Procedures, “SM cells”). SM cells constitutively express the CYP3A4 gene.

### Selection with ammonia

Cells were treated with ammonia at different concentration for 2 days to obtain ammonia-selected cells. The ammonia-selected cells were propagated for 7 days after exposure to ammonia in modified F medium^34^ until semi-confluence, and then passaged into 10-cm dishes after trypsinization with 0.25% trypsin/EDTA. If the rate of cell death after ammonia treatment was high, the medium was changed immediately.

### Hepatocytic maturation via spheroid formation

The ammonia-selected hepatocyte-like cells were detached with 0.25% trypsin/EDTA, transferred to 6-well plates at a density of 60,000 cells/well, and then cultivated for 7 or 10 days to form spheroids.

### Population doubling assay

Cells were harvested at sub-confluency and the total number of cells in each well was determined using a cell counter. Population doubling (PD) was used as the measure of cell growth. PD was calculated from the formula PD = log_2_(A/B), where A is the number of harvested cells and B is the number of plated cells^35^.

### Immunocytochemical analysis

Cells were fixed with 4% paraformaldehyde in PBS for 10 min at room temperature. After washing with PBS and treatment with 0.1% Triton X in PBS for 10 min, cells were pre-incubated with blocking buffer (5% goat serum in PBS) for 30 min at room temperature, and then exposed to primary antibodies [CYP3A4 (HL3, SANTACRUZ BIOTECHNOLOGY, sc-53850), α-Fetoprotein (R&D SYSTEMS, MAB1368), Albumin (CEDARLANE, CLFAG2140), CPS1 (Anti-CPS1 antibody, abcam, ab45956), Cytokeratin 7 (DAKO, M7018), GLUL (HPA007571, Sigma-Aldrich)] in blocking buffer overnight at 4 °C. Following washing with 0.2% PBST, cells were incubated with secondary antibodies; either goat anti-rabbit or anti-mouse IgG conjugated with Alexa 488 or 546 (1:1000) (Invitrogen) in blocking buffer for 30 min at room temperature. Then, the cells were counterstained with DAPI and mounted.

### Quantitative RT-PCR

RNA was extracted from cells using the RNeasy Plus Mini Kit (QIAGEN: 74136). An aliquot of total RNA was reverse transcribed using an oligo (dT) primer (SUPERSCRIPT III First-Strand Synthesis System, INVITROGEN). For the thermal cycle reactions, the cDNA template was amplified (QUANTSTUDIO 12K Flex Real-Time PCR System) with gene-specific primer sets (Table 1) using the Platinum Quantitative PCR SuperMix-UDG with ROX (11743-100, INVITROGEN) under the following reaction conditions: 40 cycles of PCR (95 °C for 15 s and 60 °C for 1 min) after an initial denaturation (50 °C for 2 min and 95 °C for 2 min). Fluorescence was monitored during every PCR cycle at the annealing step. The authenticity and size of the PCR products were confirmed using a melting curve analysis (using software provided by Applied Biosystems) and gel analysis. mRNA levels were normalized using ubiquitin or GAPDH as a housekeeping gene^21^.

**Table 1:** Primer pairs for RT-PCR.

Gene product	Forward and reverse primers (5′–3′)
ALB	TGGCACAATGAAGTGGGTAA
	CTGAGCAAAGGCAATCAACA
AFP	GCTTGGTGGTGGATGAAAC
	CCCTCTTCAGCAAAGCAGAC
CYP3A4	CAAGACCCCTTTGTGGAAAA
	CGAGGCGACTTTCTTTCATC
CPS1	CAAGTTTTGCAGTGGAATCG
	GGACAGATGCCTGAGCCTA
OTC	TTTCCAAGGTTACCAGGTTACAA
	CTGGGCAAGCAGTGTAAAAAT
CK7	GAGGTCACCATTAACCAGAGCC
	GCAATCTGGGCCTCAAAGATGT
UBIQUITIN	GGAGCCGAGTGACACCATTG
	CAGGGTACGACCATCTTCCAG

### Urea measurement

To determine urea secretion, supernatants of cell culture were collected from 6-well plates after 48-h culture. The concentration of secreted urea was measured by the QUANTICHROM Urea Assay Kit (BioAssay Systems) according to the manufacturer’s instructions.

## Supplementary Information

Supplementary Information 1.

Supplementary Information 2.
