# Supplementary material for: Ammonia-based enrichment and long-term propagation of zone I hepatocyte-like cells
Source: Sci Rep. 2021 May 31;11:11381. doi: 10.1038/s41598-021-90708-3 (PMC8166824; doi:10.1038/s41598-021-90708-3)
Supplement: Supplementary file 1 — Supplementary Information 1. [file 41598_2021_90708_MOESM1_ESM.pdf]

Ammonia-based enrichment and long-term propagation of zone I hepatocyte-like cells

Ruri Tsuneishi[1,2], Noriaki Saku[1], Shoko Miyata[1], Saeko Akiyama[1], Palaksha Kanive Javaregowda[1], Kenta Ite[1], Nagisa Takashima[1], Masashi Toyoda[1,3], Tohru Kimura[4], Masahiko Kuroda[5], Atsuko Nakazawa[1,6], Mureo Kasahara[7], Hidenori Nonaka[1], Akihide Kamiya[8], Tohru Kiyono[9], Junji Yamauchi[2], and Akihiro Umezawa[1*]

[1] Center for Regenerative Medicine, National Center for Child Health and Development Research Institute, Tokyo, 157-8535, Japan
[2] Laboratory of Molecular Neuroscience and Neurology, Tokyo University of Pharmacy and Life Sciences, Hachioji, Tokyo, 192-0392, Japan
[3] Research team for Geriatric Medicine (Vascular Medicine), Tokyo Metropolitan Institute of Gerontology, Tokyo, 173-0015, Japan
[4] Laboratory of Stem Cell Biology, Department of Biosciences, Kitasato University School of Science, Kanagawa 252-0373, Japan
[5] Department of Molecular Pathology, Tokyo Medical University, 6-1-1 Shinjuku, Shinjuku-ku, Tokyo, 160-8402, Japan.
[6] Saitama Children's Medical Center, Saitama, 330-8777, Japan
[7] Organ Transplantation Center, National Center for Child Health and Development, Tokyo, 157-8535, Japan
[8] Department of Molecular Life Sciences, Tokai University School of Medicine, 143 Shimokasuya, Isehara, Kanagawa, 259-1193, Japan.
[9] Project for Prevention of HPV-related Cancer, Exploratory Oncology Research and Clinical Trial Center, National Cancer Center, Chiba, 277-8577, Japan

*Correspondence should be directed to:
Akihiro Umezawa,
Center for Regenerative Medicine
National Center for Child Health and Development Research Institute
2-10-1 Okura, Setagaya,
Tokyo, 157-8535, JAPAN
Phone: +81-3-5494-7047
Fax: +81-3-5494-7048
E-mail: umezawa@1985.jukuin.keio.ac.jp

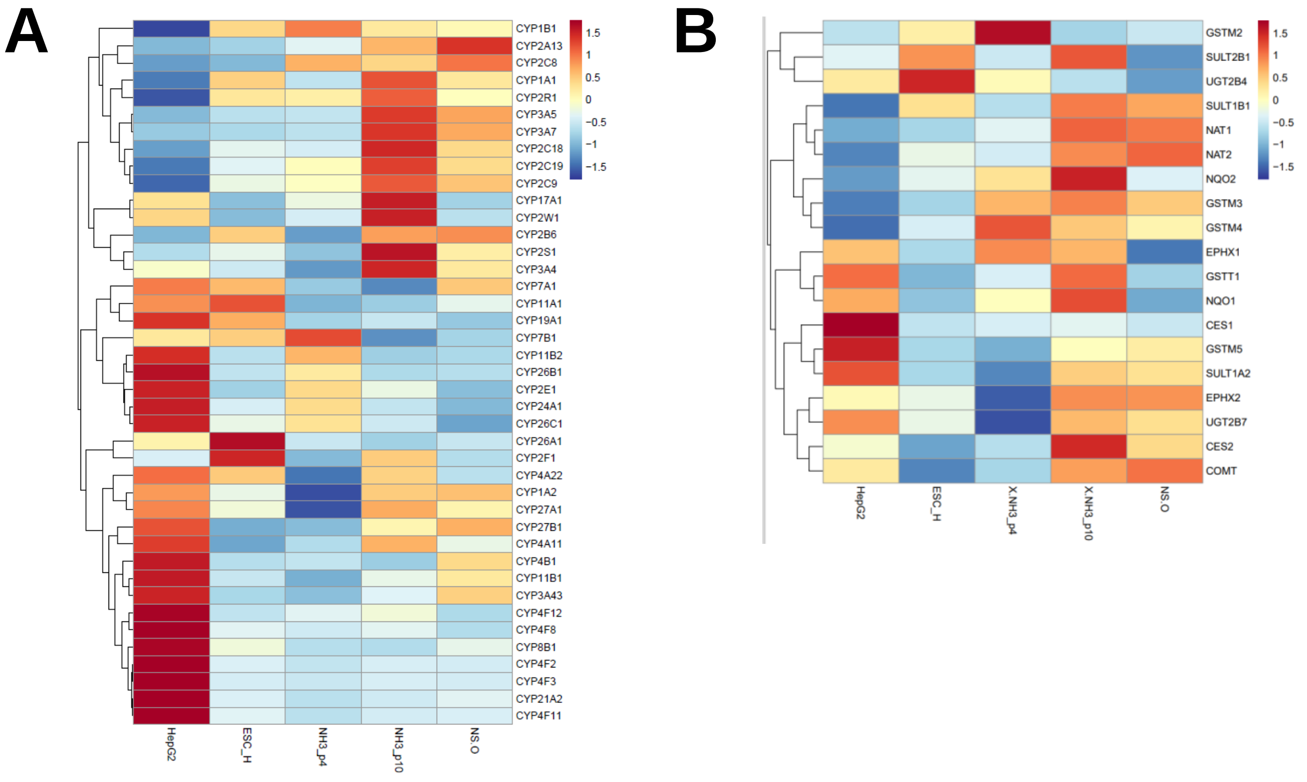

Supplementary Figure 1. Gene chip analysis of ammonia-selected ESC-derived hepatocyte-like cells.

We performed gene chip analysis of SEES2-derived hepatocyte-like cells and iPSC-O-derived hepatocytes to investigate expression of the genes for cytochrome P450 (A) and phase 2 enzymes (B). From left to right: HepG2, ESC-derived hepatocyte-like cells without ammonia selection (ESC_H), ammonia-selected ESC-derived hepatocyte-like cells at passage 4 and 10 (X.NH3_p4 and (X.NH3_p10), ammonia-selected hepatocyte-like cells generated from DILI-derived iPSC-O cells (NS.O). (A) Genes for cytochrome P450. SEES2-derived hepatocyte-like cells at passage 10 showed increased expression of the genes for CYP1A2, CYP2B6, CYP2C9, CYP2C19, CYP3A5 and CYP3A7. (B) Genes for phase II drug metabolizing enzymes such as UDP-glucuronosyltransferases (UGTs), sulfotransferases (SULTs), N-acetyltransferases (NATs), glutathione S-transferases (GSTs), thiopurine S-methyltransferase (TPMT) and catechol O-methyltransferase (COMT). Upon exposure to ammonia, SEES2-derived hepatocyte-like cells showed increased expression of the genes for COMT, EPHX1, EPHX2, SULT1B1, SULT1A2, SULT2B1, UGT2B7, CES2, GSTM3, GSTM4, GSTM5, GSTT1, NQO1, NQO2, NAT1 and NAT2, but did not increase the expression of UGT2B4, CES1, and GSTM2.
