# Supplementary material for: Ammonia-based enrichment and long-term propagation of zone I hepatocyte-like cells
Source: Sci Rep. 2021 May 31;11:11381. doi: 10.1038/s41598-021-90708-3 (PMC8166824; doi:10.1038/s41598-021-90708-3)
Supplement: Supplementary file 2 — Supplementary Information 2. [file 41598_2021_90708_MOESM2_ESM.docx]

**Ammonia-based enrichment and long-term propagation of zone I hepatocyte-like cells**

Ruri Tsuneishi^1,2^, Noriaki Saku^1^, Shoko Miyata^1^, Saeko Akiyama^1^, Palaksha Kanive Javaregowda^1^, Kenta Ite^1^, Nagisa Takashima^1^, Masashi Toyoda^1,3^, Tohru Kimura^4^, Masahiko Kuroda^5^, Atsuko Nakazawa^1,6^, Mureo Kasahara^7^, Hidenori Nonaka^1^, Akihide Kamiya^8^, Tohru Kiyono^9^, Junji Yamauchi^2^, and Akihiro Umezawa^1*^

^1^ Center for Regenerative Medicine, National Center for Child Health and Development Research Institute, Tokyo, 157-8535, Japan

^2^ Laboratory of Molecular Neuroscience and Neurology, Tokyo University of Pharmacy and Life Sciences, Hachioji, Tokyo, 192-0392, Japan

^3^ Research team for Geriatric Medicine (Vascular Medicine), Tokyo Metropolitan Institute of Gerontology, Tokyo, 173-0015, Japan

^4^ Laboratory of Stem Cell Biology, Department of Biosciences, Kitasato University School of Science, Kanagawa 252-0373, Japan

^5^ Department of Molecular Pathology, Tokyo Medical University, 6-1-1 Shinjuku, Shinjuku-ku, Tokyo, 160-8402, Japan.

^6^ Saitama Children’s Medical Center, Saitama, 330-8777, Japan

^7^ Organ Transplantation Center, National Center for Child Health and Development, Tokyo, 157-8535, Japan

^8^ Department of Molecular Life Sciences, Tokai University School of Medicine, 143 Shimokasuya, Isehara, Kanagawa, 259-1193, Japan.

^9^ Project for Prevention of HPV-related Cancer, Exploratory Oncology Research and Clinical Trial Center, National Cancer Center, Chiba, 277-8577, Japan

*Correspondence should be directed to:

Akihiro Umezawa,

Center for Regenerative Medicine

National Center for Child Health and Development Research Institute

2-10-1 Okura, Setagaya,

Tokyo, 157-8535, JAPAN

Phone: +81-3-5494-7047

Fax: +81-3-5494-7048

E-mail: umezawa@1985.jukuin.keio.ac.jp

# Supplemental Experimental Procedures

## SM cells

Cells were obtained from a patient with hepatic failure.  The cells were maintained in Dulbecco’s modified Eagle’s medium (DMEM, SIGMA D6429) supplemented with 20% FBS at 37°C in a humidified atmosphere containing 95% air and 5% CO_2_. When the cultures reached subconfluence, the cells were harvested with Trypsin-EDTA solution (cat# 23315, IBL CO., Ltd, Gunma, Japan), and re-plated at a density of 5 x 10^5^ cells in a 100-mm dish. Medium changes were carried out twice a week thereafter. iPSCs were generated from the patient-derived cells through reprogramming by Sendai virus infection-mediated expression of OCT4/3, SOX2, KLF4, and c-MYC [^1^](https://paperpile.com/c/nxG5bV/RBAZ), and maintained on irradiated MEFs [^2,3^](https://paperpile.com/c/nxG5bV/rMQfz+yquYk). For differentiation, iPSCs (1 x 10^4^/well) were dissociated into single cells with Accutase (Thermo Scientific, MA, USA) to generate embryoid bodies (EBs) after exposure to the ROCK inhibitor (Y-27632: A11105-01, Wako, Japan), and cultivated in the 96-well plates in the EB medium [76% KNOCKOUT DMEM, 20% KNOCKOUT Serum Replacement (Life Technologies, CA, USA), 2 mM GlutaMAX-I, 0.1 mM NEAA, Pen-Strep, and 50 µg/mL l-ascorbic acid 2-phosphate (Sigma-Aldrich, St. Louis, MO, USA)] for 10 days. The EBs were transferred to the 24-well plates coated with collagen type I and III (NMP Collagen PS, Nippon Ham Co., Ltd., Osaka, Japan), and cultivated in XF32 medium [85% KNOCKOUT DMEM, 15% KNOCKOUT Serum Replacement XF CTS (XF-KSR; Life Technologies), 2 mM GlutaMAX-I, 0.1 mM NEAA, Pen-Strep, 50 µg/mL l-ascorbic acid 2-phosphate (Sigma-Aldrich, St. Louis, MO, USA), 10 ng/mL heregulin-1β (recombinant human NRG-beta 1/HRG-beta 1 EGF domain; R&D Systems, Minneapolis, MN, USA), 200 ng/mL recombinant human IGF-1 (LONG R3-IGF-1; Sigma-Aldrich), and 20 ng/mL human bFGF (Life Technologies)] for 14 to 35 days. The differentiated cells were immortalized by infection with the lentiviral vector plasmids CSII-CMV-Tet-Off, CSII-TRE-Tight-cyclin D1, CSII-TRE-Tight-CDK4R24C, and CSII-TRE-Tight-TERT [^4,5^](https://paperpile.com/c/nxG5bV/74X4M+XjHpe). EpCAM-positive cells were isolated as described below after the immortalization from a mixed population. The immortalized cells were maintained in the modified F-medium at 37°C in a humidified atmosphere containing 95% air and 5% CO_2_ [^6,7^](https://paperpile.com/c/nxG5bV/HU2S+h3OV). EpCAM-positive cells were isolated from the differentiated endodermal cells using a magnetic cell sorting kit (MACS; Miltenyi Biotec K.K. Cologne, Germany) with the CD326 (EpCAM) MicroBeads (cat# 130-061-101, Miltenyi Biotec), according to the manufacturer’s instructions. The magnetically labeled EpCAM-positive cells were eluted as a positively selected cell fraction.  We designated one of the immortalized EpCAM-positive cells as SM cells.

## Microarray analysis

Microarray analysis including total RNA isolation was performed at DNA Chip Research (Japan). Briefly, total RNA was isolated using miRNeasy mini kit (QIAGEN). RNA samples were labeled and hybridized to a SurePrint G3 Human GE microarray 8 x 60K Ver3.0 (Agilent), and the raw data were normalized using the 75-percentile shift. Unsupervised clustering was performed with selected genes using the R package (pheatmap, function: pheatmap()).

# References

1. [Nishimura, K. *et al.* Development of defective and persistent Sendai virus vector: a unique gene delivery/expression system ideal for cell reprogramming. *J. Biol. Chem.* **286**, 4760–4771 (2011).](http://paperpile.com/b/nxG5bV/RBAZ)

2. [Makino, H. *et al.* Mesenchymal to embryonic incomplete transition of human cells by chimeric OCT4/3 (POU5F1) with physiological co-activator EWS. *Exp. Cell Res.* **315**, 2727–2740 (2009).](http://paperpile.com/b/nxG5bV/rMQfz)

3. [Nishino, K. *et al.* DNA methylation dynamics in human induced pluripotent stem cells over time. *PLoS Genet.* **7**, e1002085 (2011).](http://paperpile.com/b/nxG5bV/yquYk)

4. [Shiomi, K. *et al.* CDK4 and cyclin D1 allow human myogenic cells to recapture growth property without compromising differentiation potential. *Gene Ther.* **18**, 857–866 (2011).](http://paperpile.com/b/nxG5bV/74X4M)

5. [Yugawa, T. *et al.* Regulation of Notch1 gene expression by p53 in epithelial cells. *Mol. Cell. Biol.* **27**, 3732–3742 (2007).](http://paperpile.com/b/nxG5bV/XjHpe)

6. [Yachida, S. *et al.* Genomic Sequencing Identifies ELF3 as a Driver of Ampullary Carcinoma. *Cancer Cell* **29**, 229–240 (2016).](http://paperpile.com/b/nxG5bV/HU2S)

7. [Liu, X. *et al.* ROCK inhibitor and feeder cells induce the conditional reprogramming of epithelial cells. *Am. J. Pathol.* **180**, 599–607 (2012).](http://paperpile.com/b/nxG5bV/h3OV)
